# Comparative Prey Spectra Analyses on the Endangered Aquatic Carnivorous Waterwheel Plant (*Aldrovanda vesiculosa*, Droseraceae) at Several Naturalized Microsites in the Czech Republic and Germany

**DOI:** 10.1093/iob/oby012

**Published:** 2019-03-25

**Authors:** M Horstmann, L Heier, S Kruppert, L C Weiss, R Tollrian, L Adamec, A Westermeier, T Speck, S Poppinga

**Affiliations:** 1 Department of Animal Ecology, Evolution and Biodiversity, Ruhr-University Bochum, Universitätsstraße 150, D-44780, Bochum, Germany; 2 Friday Harbor Laboratories, University of Washington, 620 University Road, WA 98250, USA; 3 Institute of Botany of the Czech Academy of Sciences, Dukelská 135, CZ-379 82, Třeboň, Czech Republic; 4 Plant Biomechanics Group, Botanic Garden, University of Freiburg, Schänzlestraße 1, D-79104, Freiburg im Breisgau, Germany; 5 Freiburg Center for Interactive Materials and Bioinspired Technologies (FIT), University of Freiburg, Georges-Koehler-Allee 105, D-79110, Freiburg im Breisgau, Germany; 6 Freiburg Materials Research Center (FMF), University of Freiburg, Stefan-Meier-Straße 21, D-79104 Freiburg im Breisgau, Germany

## Abstract

The critically endangered carnivorous waterwheel plant (*Aldrovanda vesiculosa*, Droseraceae) possesses underwater snap traps for capturing small aquatic animals, but knowledge on the exact prey species is limited. Such information would be essential for continuing ecological research, drawing conclusions regarding trapping efficiency and trap evolution, and eventually, for conservation. Therefore, we performed comparative trap size measurements and snapshot prey analyses at seven Czech and one German naturalized microsites on plants originating from at least two different populations. One Czech site was sampled twice during 2017. We recorded seven main prey taxonomic groups, that is, Cladocera, Copepoda, Ostracoda, Ephemeroptera, Nematocera, Hydrachnidia, and Pulmonata. In total, we recorded 43 different prey taxa in 445 prey-filled traps, containing in sum 461 prey items. With one exception, prey spectra did not correlate with site conditions (e.g. water depth) or trap size. Our data indicate that *A. vesiculosa* shows no prey specificity but catches opportunistically, independent of prey species, prey mobility mode (swimming or substrate-bound), and speed of movement. Even in cases where the prey size exceeded trap size, successful capture was accomplished by clamping the animal between the traps’ lobes. As we found a wide prey range that was attracted, it appears unlikely that the capture is enhanced by specialized chemical- or mimicry-based attraction mechanisms. However, for animals seeking shelter, a place to rest, or a substrate to graze on, *A. vesiculosa* may indirectly attract prey organisms in the vicinity, whereas other prey capture events (like that of comparably large notonectids) may also be purely coincidental.

## Synopsis

Die stark gefährdete karnivore Wasserfalle (*Aldrovanda vesiculosa*, Droseraceae) bildet Unterwasser-Schnappfallen zum Fang von aquatischen Kleinstlebewesen aus, jedoch wurde das Beutespektrum bislang noch nicht ausreichend untersucht. Eine genaue Kenntnis der Zusammensetzung der Beutetiere ist jedoch sehr wichtig, um Fragestellungen bzgl. der Ökologie dieser fleischfressenden Pflanze, der Effizienz und Evolution ihrer komplexen Fallen, sowie hinsichtlich Naturschutzmaßnahmen beantworten zu können. Hierfür wurden bei künstlich wieder angesiedelten, etablierten Vorkommen in einem deutschen und in sieben tschechischen Kleinst-Habitaten die Fallengrößen gemessen und Falleninhalte vergleichend analysiert. Die untersuchten Pflanzen entstammen dabei aus mindestens zwei mitteleuropäischen Ursprungspopulationen. Ein tschechischer Standort wurde im Laufe des Jahres 2017 zweimal beprobt und hinsichtlich der Saisonalität ausgewertet. Es wurden sieben Hauptgruppen an Beutetieren erfasst: Cladocera, Copepoda, Ostracoda, Ephemeroptera, Nematocera, Hydrachnidia und Pulmonata. Insgesamt konnten in den 445 untersuchten Fallen, die insgesamt 461 gefangene Tiere enthielten, 43 Beutetaxa bestimmt werden. Die Beute-Spektren korrelierten dabei (mit einer Ausnahme) nicht mit den Standortbedingungen (z.B. Wassertiefe) oder der Größe der Fallen. Unsere Daten zeigen, dass *A. vesiculosa* keine Beutespezifität aufweist, sondern opportunistisch Beute fängt, unabhängig von Taxonomie, Fortbewegungsmodus (schwimmend oder kriechend) oder der Bewegungsgeschwindigkeit der Beute. Erfolgreiche Fänge durch Einklemmen des Tieres zwischen den Fallenhälften wurden auch bei Beutetieren mit Körpermaßen beobachtet, die die Größe der Falle deutlich überschritten. Durch die hohe Diversität an Beutetaxa erscheint es unwahrscheinlich, dass es spezielle chemische oder Mimikry-basierende Faktoren zur Anlockung gibt. Die Pflanze könnte jedoch als Ruhe- oder Ansitzplatz, sowie als Substrat zum Abweiden (z.B. von Algen) dienen und so indirekt Beuteorganismen in der Umgebung anlocken. Die Beutefangereignisse, wie z.B. die von vergleichsweise großen Notonectiden, könnten jedoch auch rein zufällig sein.

La planta carnívora (*Aldrovanda vesiculosa*, Droseraceae), clasificada en peligro crítico de extinción, posee trampas submarinas para la captura de pequeños animales acuáticos, pero el conocimiento exacto de las especies usadas como presas es limitado. Dicha información sería esencial para continuar la investigación ecológica, sacar conclusiones con respecto a la eficiencia de captura y la evolución de la estructura trampa, y finalmente, para problemas de conservación. Por lo tanto, realizamos mediciones comparativas del tamaño de la trampa y un análisis de referencia de las presas en siete micrositios naturalizados checos y uno alemán en plantas que se originan en al menos dos poblaciones diferentes. Uno de los sitios checos fue muestreado dos veces durante el año 2017. Se registraron siete grupos taxonómicos de presas como Cladocera, Copepoda, Ostracoda, Ephemeroptera, Nematocera, Hydrachnidia y Pulmonata. En total, registramos 435 taxones diferentes en 445 trampas llenas de presas, que contienen en total 461 presas. Los espectros de presa no se correlacionaron con las condiciones del sitio (por ejemplo, la profundidad del agua) o el tamaño de la trampa. Nuestros datos indican que *A. vesiculosa* no muestra preferencia por un tipo de presa, sino que captura de manera oportunista, independientemente de la especie, el modo de movilidad (móvil o fija al sustrato) y la velocidad de movimiento de la presa. Incluso en los casos en que el tamaño de la presa excedía el tamaño de la trampa, la captura exitosa se logra sujetando al animal entre los lóbulos de la trampa. Como encontramos un amplio rango de presas atraídas, parece poco probable que las capturas sean mejoradas por un mecanismo especializado de atracción basado en químicos o mimetismo. Sin embargo, para los animales que buscan refugio, un lugar para descansar o un sustrato para pastar, *A. vesiculosa* puede atraer indirectamente a las presas en las proximidades, mientras que otras presas capturadas (como los notónectidos que son similarmente grandes a las plantas) pueden ser pura coincidencia. (translation by Camilo Alfonso)

Kriticky ohrožená masožravá aldrovandka měchýřkatá (*Aldrovanda vesiculosa*, Droseraceae) používá vodní chňapací pasti pro chytání malých vodních živočichů, ale přesná znalost o druzích kořisti je omezená. Taková informace je nezbytná pro pokračující ekologický výzkum, pro formulování závěrů týkajících se účinnosti chytání a evoluce pasti a případně i pro ochranářská témata. Provedli jsme tudíž srovnávací měření velikosti pastí a analýzy ulovené kořisti na sedmi českých a jednom německém naturalizovaném mikrostanovišti na rostlinách pocházejících alespoň ze dvou odlišných populací. Jedno české mikrostanoviště bylo sledováno dvakrát v roce 2017. Zaznamenali jsme sedm hlavních taxonomických skupin (Cladocera, Copepoda, Ostracoda, Ephemeroptera, Nematocera, Hydrachnidia a Pulmonata). Celkem jsme zaznamenali 43 různých taxonů kořisti v 445 pastech s ulovenou kořistí obsahujících celkem 461 jedinců kořisti. Spektra kořisti nekorelovala se stanovištními podmínkami (např. hloubka vody) nebo velikostí pastí. Výsledky naznačují, že aldrovandka nejeví žádnou specificitu kořisti, ale loví oportunisticky, tj. nezávisle na druzích kořisti, pohyblivosti kořisti (plovoucí nebo vázaná k substrátu) nebo rychlosti pohybu. I v případech, kdy velikost kořisti převyšovala velikost pasti, kořist byla úspěšně ulovena zachycením mezi laloky pasti. Protože jsme zjistili široké spektrum ulovené kořisti, je nepravděpodobné, že chytání je podporováno specializovaným chemickým nebo na mimikry založeným mechanismem atrakce. Avšak živočichy hledající úkryt nebo místo k odpočinku anebo potravní substrát může aldrovandka nepřímo lákat ve své blízkosti, kdežto ulovení jiné kořisti (např. srovnatelně velkých znakoplavek) může být čistě náhodné.

## Introduction

The waterwheel plant (*Aldrovanda vesiculosa* L., Droseraceae) is a rootless, free-floating aquatic carnivorous plant. It floats beneath the water surface of shallow, nutrient-poor, dystrophic (humic) standing waters in Australia, Africa, Asia, and continental Europe (Lloyd 1942; Adamec 1995; Cross 2012; Brewer and Schlauer 2018). Temperate populations overwinter as turions (Cross 2012). Owing to its strict stenotopic character and very low competitive ability, its geographical distribution is limited and irregular (Adamec 1995) so that populations are typically small and fragmented (Cross 2012; Cross et al. 2015). In addition, due to habitat loss, the waterwheel plant was declared as critically endangered and has been anthropogenically introduced to suitable sites (Akeret 1993; Adamec and Lev 1999; Adamec 2005; Lamont et al. 2013; Cross et al. 2015; Floyd et al. 2015; Gorissen 2015).

The waterwheel plant develops snap-traps (Lloyd 1942) for the capture of prey (predominantly zooplankton) as a substantial nutrient supply (Adamec et al. 2010; Adamec 2018a; Cross et al. 2015). These snap traps are 2.5–6 mm long and function in the aquatic medium (Ashida 1934; Poppinga et al. 2018; Westermeier et al. 2018). They are arranged in regularly interspaced leaf whorls along the stem, giving rise to the common name “waterwheel”. This specific snap-trap arrangement and orientation is hypothesized to increase the capture radius of the plant for potential prey (Cross 2012). Long bristles situated near each snap-trap are speculated to prevent objects other than prey (e.g. detritus) from accidentally entering the snap-traps. Furthermore, they could guide substrate-dwelling animals (e.g. small crustaceans) toward the snap-trap (Cross 2012; Poppinga et al. 2018). Trigger hairs inside the snap-trap were discussed to mimic filamentous algae in order to lure grazing crustaceans into the snap-trap (Schell 2003). Mechanical stimuli by animals entering the snap-trap entail rapid trap closure within 20–50 ms, followed by trap narrowing, and digestion of prey within 3–10 days, until the snap-trap reopens (Ashida 1934; Poppinga and Joyeux 2011; Westermeier et al. 2018). One trap can even capture multiple prey items simultaneously (Cross 2012).

The snap-trap functioning and prey capture mechanism in *A. vesiculosa* have been well described (Ashida 1934; Iijima and Sibaoka 1983; Poppinga and Joyeux 2011; Westermeier et al. 2018). Despite some records published (Darwin 1875; Cross 2012; Darnowski et al. 2018) the prey spectrum of this carnivorous plant is still disputed. It remains speculative whether *A. vesiculosa* is a generalist or specialist consumer. Moreover, whether *Aldrovanda* possesses prey-attracting features is also unknown (cf. Darnowski et al. 2018; Horner et al. 2018). Akeret (1993) presents the most detailed prey spectrum analysis of *Aldrovanda* to date, based on a snapshot sampling of 252 traps from five plants collected from a Swiss extracted fen pool in August 1993: of the 32 prey animals found, 12 (37.5%) were members of Cladocera, 9 (28.12%) Diptera larvae, 5 (15.63%) Ostracoda, 2 (6.25%) Gastropoda, 2 (6.25%) Copepoda, and 2 (6.25%) Ephemeroptera larvae. Many traps also contained unidentifiable, half-digested prey. The author attributes only low prey capture efficiency to *Aldrovanda* due to the observation of a low number of traps with prey (11.5% in total) in direct comparison to co-occurring carnivorous *Utricularia australis* (Lentibulariaceae, with suction traps). At two Czech artificial *Aldrovanda* sites, which were used also in the present study, only about 5–8% of *Aldrovanda* traps and about 1–4% of the traps of co-occurring *U. australis*, captured prey (Adamec and Kovářová 2006). In contrast, Cross et al. (2015) found in their field investigation that up to 40% of the maximum 200 traps per *Aldrovanda* plant contained (unidentified) prey. By feeding cultivated *Aldrovanda* with zooplankton mixtures, Schell (2003) and Adamec et al. (2010) noted that ostracods were predominantly captured.

Other carnivorous plants, either with the same trap type (*Dionaea muscipula*) or plants that share similar habitats (aquatic bladderworts of *Utricularia* sect. *Utricularia*), have been investigated in more detail with respect to their prey spectra. *Dionaea* attracts its prey with visual and olfactory signals (reviewed by Horner et al. 2018), and it has been hypothesized (Darwin 1875) and later modeled (Gibson and Waller 2009; Lehtinen 2018) that it selectively captures comparably large prey items, assuming that the costs of capturing small animals exceed the respective benefits. However, this was not supported by field observations (Hutchens and Luken 2009, 2015) showing that the prey capture is opportunistic rather than selective, that is, large insects were not preferentially captured over smaller prey items, with the predominant prey consisting of smaller sized spiders, ants, and beetles.

Aquatic bladderworts (*Utricularia* sect. *Utricularia*) capture their prey with millimeter-sized, ultra-fast suction traps (so-called bladders) which function in the millisecond regime (Vincent et al. 2011; Poppinga et al. 2017). In contrast to *A. vesiculosa*, whose traps stay closed during prey digestion (Lloyd 1942), *Utricularia* is able to continue catching additional animals with the same trap even during digestion (Poppinga et al. 2016b). Aquatic *Utricularia* typically capture a wide range of small ciliates, rotifers, nematodes, tardigrades, crustaceans (especially cladocerans, copepods, and ostracods), mites, and gastropods as well as, occasionally, comparably large animals like Nematocera and Odonata larvae, salamanders, or even young fish (reviewed by Poppinga et al. 2016b; Darnowski et al. 2018). *Utricularia australis*, the bladderwort species which co-occurred the *Aldrovanda* population examined by Akeret (1993), captures predominantly ciliates, cladocerans, copepods, rotifers, and insect larvae, as reported from field investigations in Germany (Mette et al. 2000; Alkhalaf et al. 2009).

To determine the prey spectrum of *A. vesiculosa*, we conducted comparative prey spectra analyses at seven naturalized *Aldrovanda* sites in the Czech Republic and at one site in Germany. The recorded prey species were taxonomically analyzed and checked for possible provenance and at one site for season-specific (natural) occurrences. We also recorded snap-trap sizes and correlated these with prey species size. With this we were able to determine the degree of prey preference of *Aldrovanda.*

## Methods

### Sampling sites

**Fig. 1:**
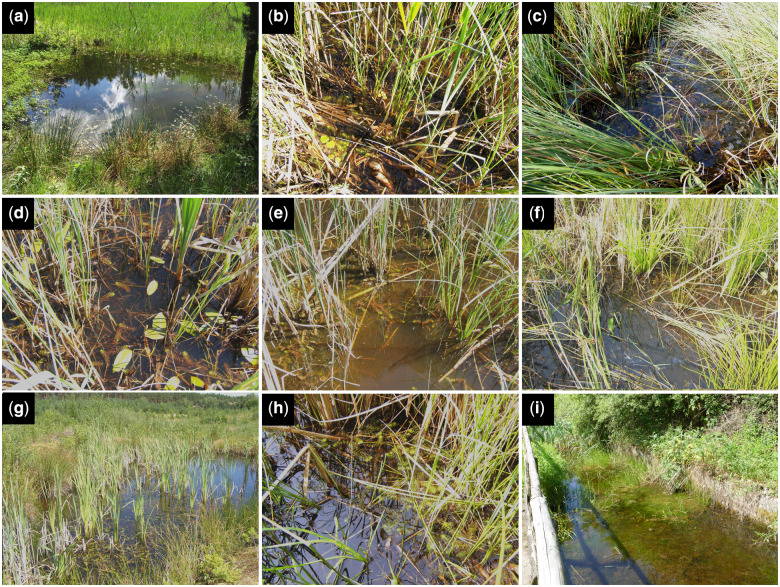
The sampled, naturalized *A. vesiculosa* sites from Czech Republic (CZ) (a‑h) and Germany (GER) (i). (a) Branná sand-pit (sampling event CZ1). (b) Karštejn fen lake (CZ2). (c) Karštejn fen lake (CZ3). (d) Karštejn fen lake, main microsite (CZ4). (e) Ptačí blato 1st lagoon, 1st sampling (CZ5_June_). (f) Ptačí blato 1st lagoon, 2nd sampling (CZ5_August_). (g) Suchdol nad Lužnicí sand-pit (CZ6). (h) Ptačí blato 4th lagoon (CZ7). (i) Concrete basin at the Nature Protection Area Wahner Heide (GER).

Eight microsites (seven in the Třeboňsko Protected Landscape Area in South Bohemia, Czech Republic, one in North Rhine-Westphalia, Germany) with introduced *A. vesiculosa* plants were sampled in total (Fig. 1). All sampling sites and sampling events (CZ1-7, GER) are described in detail below. The Czech “Ptačí blato 1st lagoon” site was sampled twice during the 2017 growth season (CZ5_June_ and CZ5_August_). The Czech *Aldrovanda* plants originate from Lake Długie in E Poland (sites CZ1-5, 7) or from Baláta-tó Lake in SW Hungary (CZ6). For more details on plant origin, see Elansary et al. (2010), and for GPS and water chemistry, see Adamec (2005, 2009), Adamec and Kovářová (2006), and Cross et al. (2016). There was a significant low water level at sites CZ2, 3, 5, during the time of sampling. At all sampling sites, *Aldrovanda* traps with captured prey were collected on warm and sunny days (afternoon air temperature 28–30°C) between 10:00 and 15:00 of local time.

Water chemistry and temperature at the German site were analyzed using a multimeter for pH, O_2_ saturation, temperature, and electrical conductivity (Multi 3420 SET G, WTW GmbH, Weilheim, Germany). The parameters for the Czech sites were measured using ECTestr 11+ (EU), pH electrode, and a pH meter (Hanna, Portugal).

#### Czech sites (Třeboňsko)

CZ1 (Branná sand-pit, Fig. 1a): A small dystrophic and slightly eutrophic sand-pit pool near Branná with a water depth of 25–40 cm. The dominant accompanying vegetation consisted of *Comarum palustre*, *Juncuseffusus*, and *Spirogyra* sp. The *Aldrovanda* plants were 8–12 cm long and occurred in sparse density. More than 50 full traps from 15 plants were collected on June 10, 2017. The electrical conductivity of the water was 7.9 mS m^−1^.

CZ2 (Karštejn fen lake, Fig. 1b): A shallow dystrophic and mesotrophic pool in a dense stand of *Carex rostrata* with *Chara fragilis* in the complex of the excavated fen Kramářka in the Nežárka river floodplain at a water depth of 10–15 cm. The *Aldrovanda* plants were 6–10 cm long and occurred in moderate density. More than 50 filled traps were collected from 16 plants on June 11, 2017.

CZ3 (Karštejn fen lake, Fig. 1c): A dense shallow dystrophic and mesotrophic reed stand in the complex of the excavated fen Kramářka at a water depth of 5–12 cm. The dominant accompanying vegetation consisted of *Carex rostrata*, *Comarum palustre*, *Hydrocharis morsus-ranae*, and *Phragmites australis*. The *Aldrovanda* plants were 6–12 cm long and occurred in moderate density. More than 50 filled traps were collected from 28 plants on June 11, 2017.

CZ4 (Karštejn fen lake, main microsite, Fig. 1d): Central part of the dystrophic and mesotrophic fen lake with *Nymphaea candida* in the complex of the excavated fen Kramářka at a water depth of 20–30 cm. The dominant accompanying vegetation consisted of *Carex rostrata, Nymphaea candida, Phragmites australis*, and *Typha latifolia*. The *Aldrovanda* plants were 6–12 cm long and occurred densely. Twenty-five filled traps were collected from 30 plants on June 11, 2017. Plants at this microsite were slightly covered with precipitated humic acids on their surface. The water conductivity was 16.0 mS m^−1^. For details on water chemistry, see Cross et al. (2016).

CZ5_June_ (Ptačí blato 1st lagoon, Fig. 1e): First dystrophic–eutrophic lagoon adjacent to Ptačí blato fishpond at a water depth of 10–15 cm. The dominant accompanying vegetation consisted of *Carex acuta, Juncus effusus, Phragmites australis, Typha angustifolia*, and *Spirogyra* sp. The robust *Aldrovanda* plants were 8–18 cm long and occurred in sparse density. More than 50 filled traps were collected from 24 plants on June 11, 2017. The water conductivity was 11.2 mS m^−1^.

CZ5_August_ (Ptačí blato 1st lagoon, Fig. 1f): The same microsite as CZ5_June_. The *Aldrovanda* plants were 7–12 cm long and occurred in moderate density. More than 50 filled traps were collected from 10 plants on August 27, 2017. The water conductivity was 14.7 mS m^−1^ and pH 6.42.

CZ6 (Suchdol nad Lužnicí sand-pit, Fig. 1g): A small, slightly dystrophic and mesotrophic pool at a water depth of 5–40 cm. The dominant accompanying vegetation consisted of *Juncus bulbosus, Juncus effusus, Potamogeton natans, Typha angustifolia*, and *Utricularia bremii*. The *Aldrovanda* plants were 6–10 cm long and occurred in moderate density. More than 60 filled traps were collected from 11 plants on August 5, 2017. The water conductivity was 25.6 mS m^−1^.

CZ7 (Ptačí blato 4th lagoon, Fig. 1h): Fourth dystrophic–eutrophic lagoon adjacent to Ptačí blato fishpond at a water depth of 5–10 cm. The dense dominant accompanying vegetation consisted of *Typha latifolia, Phragmites australis, Juncus effusus*, and *Carex* spp. and shaded considerably the submerged vegetation. The *Aldrovanda* plants were 6–15 cm long and occurred in moderate density. More than 50 filled traps were collected from nine plants on August 27, 2017. The water conductivity was 13.8 mS m^−1^ and pH 6.24.

At all Czech microsites except for CZ1, the *Aldrovanda* population size exceeded about 500–1000 plants at the sampling time, while that at CZ1 was only 100–200 plants.

#### German site

GER (Wahner Heide, Fig. 1i): The German site in the Nature Protection Area Wahner Heide near Bonn is a 10 m long, 4 m wide, and 70 cm deep concrete basin formerly used as military tank wash (“Panzerwaschanlage”) within a former military area. The introduced population of *Aldrovanda* is of an unknown origin. We collected 34 traps from 15 plants (ca. 2–8 cm long) on May 29, 2017 at around 12:00. At this time, the water temperature was 26.1°C, the water conductivity was 21.5 mS m^−1^, the oxygen saturation was 178%, and pH 9.14. The water-filled basin was densely filled with vegetation, and the accompanying dominant plants were *Ceratophyllum demersum*, *Chara* sp*., Eleocharis palustris, Potamogeton* sp*., Typha latifolia*, and *Utricularia australis.* Water chemistry and dense vegetation indicate unfavorable growing conditions for *Aldrovanda* (cf. Adamec 2018b), which may explain the limited number of plants found (15 in May 2017) within this formerly much larger population (Gorissen 2015).

### General sampling procedures

*Aldrovanda* plants were taken from the habitats and transferred into petri dishes filled with water. We inspected the plants for closed traps with dark biomass inside. We sampled only traps from the whorls 3–8 counted from the apex at the German site and whorl 2–12 at the Czech sites, to guarantee that compared traps were more or less of the same size. Filled snap-traps were dissected with small forceps and transferred into small glass vials or Eppendorf tubes filled with 70% ethanol at the German site and with 4% formaline at the Czech sites. All traps were dissected from various plants and various whorls to reduce sampling bias. After sampling, the plants were transferred back to the habitats.

Traps were opened using dissection needles and their content was analyzed and recorded with an Olympus SZX 16 stereo microscope and a colorview III camera (Olympus, Tokio, Japan). Species identification was performed according to Scourfield and Harding (1994), Streble and Krauter (2006), and Schaefer (2009).

### Data analysis

In total, we collected 742 traps from all field sites. We determined snap-trap lengths in 722 snap-traps by measuring the respective midrib lengths, excluding their apical parts which protrude from the snap-trap cavities. From these 722 measured snap-traps, 445 were analyzed for prey items. The other traps were either empty or detritus-filled. All data were analyzed in a site-specific manner. For comparative analyses, we furthermore pooled all Czech sites (CZ_Total_) to allow for a geographical comparison between Germany and the Czech Republic, and furthermore all Czech sites sampled in June for a comparison of traps sampled in May/June in the Czech Republic and Germany. All statistical investigations and plotting were carried out in GNU R x64 v. 3.4.2 (R Development Core Team 2008). In addition, we used the R packages “ggplot2” (Wickham 2009) and “ggpubr” (Kassambara 2017). Within ggpubr, a Wilcoxon-test was carried out to compare the sizes of traps from different time points.

After taxonomically identifying the prey, their movement types were assigned based on the ecology of the found organisms. For the calculation of correlations and evaluation of taxa and movement groups per trap size class, we introduced a continuous spectrum according to the size measurements: XS (1.90–2.49 mm), S (2.50–2.99 mm), M (3.00–3.49 mm), L (3.50–3.99 mm), and XL (4.00–4.49 mm). Prey items without any swimming ability (e.g. snails) were considered “substrate-bound”, and animals with swimming abilities that are mostly bound to the substrate we call “grazer” (e.g. Ostracoda). The planktonic prey organisms were distinguished into slow and fast swimmers, where slow swimmers ranged up to 25 mm s^−1^, and animals with at least the ability to perform quick swimming movements like copepods were considered fast swimmers. For the analysis of trap size *versus* prey size, we conducted a correlation analysis based on a linear model calculating the coefficient of determination *R*^2^ and the respective *p*-value. Due to the fact that accurate size measurements of the prey items were only possible for undigested prey, we categorized the prey items with ascending average size, where “size” is the length measured along the longitudinal body axis. We conducted so based on the literature for average sizes of species (McCafferty and Provonsha 1998; Forró et al. 2008; Di Sabatino et al. 2008) as well as on personal experience in prey items with no published average size. We then tested for differences in prey spectra across the trap sizes using the Kruskal–Wallis test.

To test for possible correlations between trap content and water depth of the microsites, we calculated Spearman correlations with their respective correlation coefficients and *p*-values. Therefore, we defined a water depth estimation for every site by calculating the mean of maximum and minimum depth and checked for a correlation with taxonomic groups of prey items that were observed at every sampling site. Whereas *A. vesiculosa* is a rootless, free-floating plant and occurs only in the uppermost portion of the water column, depth selection preferences determine prey species distribution across the whole column of water.

## Results

### Trap sizes

By analyzing all site-specific trap sizes, we found a huge variance with trap lengths varying between 1.92 mm and 4.49 mm. The smallest traps were found at the Wahner Heide site with a 2.81 mm median trap size. The largest median for trap size was found during the sampling event CZ5_June_ with 3.62 mm. The trap sizes within all individual microsites were distributed uniformly, while spanning site-individual ranges of about 2.00 mm, with the exception of the sampling from Wahner Heide, where we found a narrower range of about 1.00 mm. At this site, as well as during the sampling event CZ5_June_, many traps were found to have sizes close to the median. The median trap size considering samplings from every microsite is 3.31 mm.

**Fig. 2:**
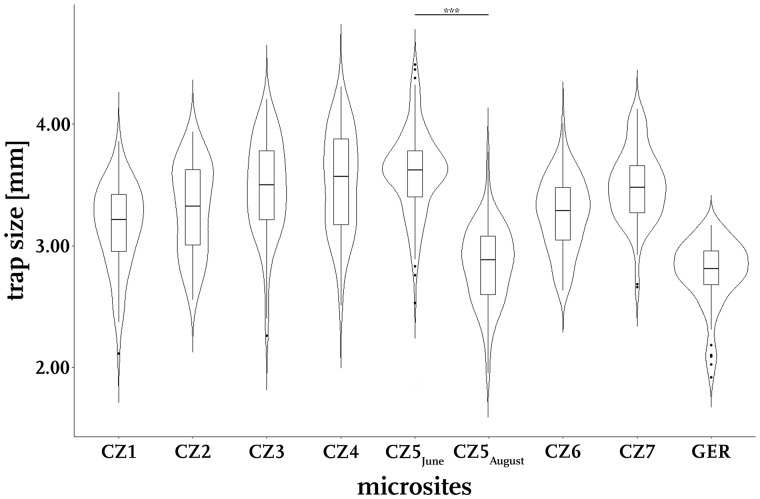
Trap size distributions at the investigated microsites. Trap sizes vary clearly between and within the investigated microsites, often spanning a range of more than 2.00 mm. The Czech site “Ptačí blato 1st lagoon” was sampled twice during the year 2017 (CZ5_June_ and CZ5_August_). In June, the traps were significantly larger than in August (Wilcoxon-test, W=10,555, n_June_=127, n_August_=88, *p* < 0.001).

We found significant differences in trap sizes from samples collected in June (CZ5_June_) and August (CZ5_August_) from the Ptačí blato 1st lagoon. Earlier traps were larger with a median length of 3.62 mm, whereas later traps were found to have a median length of 2.89 mm (Fig. 2), with a statistically significant difference (*p *<* *0.001, Wilcoxon-test, n_June_ = 127, n_August_ = 88).

### Prey spectra

**Fig. 3:**
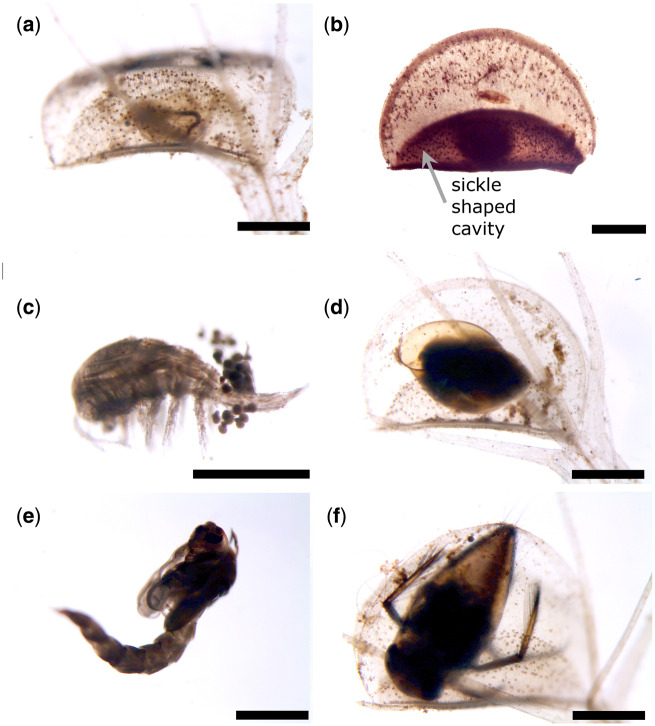
Representative prey content found in snap-traps of *A. vesiculosa*. (a) *Simocephalus expinosus* (Daphniidae). (b) Copepoda. (c) Copepoda. (d) Physidae (Mollusca). (e) Chironomidae (pupa). (f) *Notonecta* spec. (Heteroptera). In (b), the sickle shaped cavity resulting from a trap narrowing motion after prey capture is indicated (it can also be seen in (a), (d) and (f)). The scale bar is 1 mm.

**Fig. 4:**
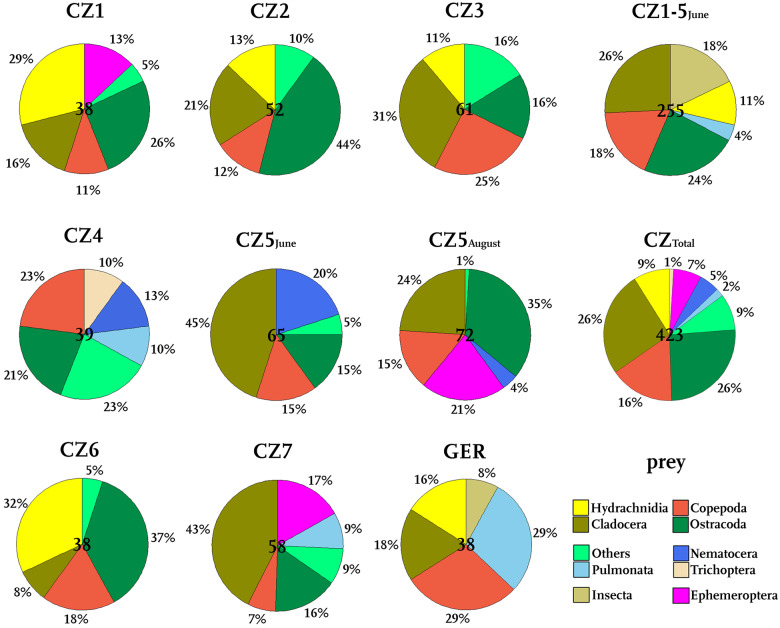
Prey spectra at all investigated microsites. Given are the sampling events (CZ1‑7, GER) and combined prey spectra (CZ_Total_, CZ1‑5_June_), the relative amounts of prey taxonomic groups and the sample size (central number in the pie chart).

**Fig. 5:**
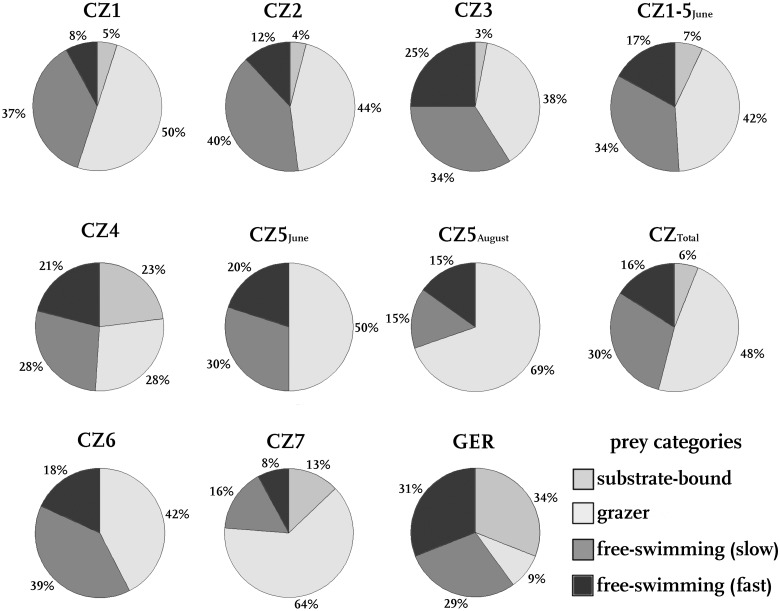
Prey organisms with different movement types per microsite. Given are the sampling microsites (CZ1‑7, GER) and combined prey spectra (CZ_Total_, CZ1‑5_June_) and the relative amounts of prey movement types.

In total, we recorded 461 prey items from 43 different taxa in 445 prey-filled traps. The diversity of prey items in the investigated snap-traps is irrespective of the sampling site (Figs. 3 and 4) and ranges from Cladocera (Fig. 3a), Copepoda (Fig. 3b, c) and Ostracoda to insect larvae (Ephemeroptera, Nematocera), Pulmonata (Fig. 3d) and Hydrachnidia. Among these, water mites represented the smallest prey (0.67 mm). The longest prey item found was a chironomid pupa (Fig. 3e), which was (with prostrated abdomen) 4.32 mm long and about 1.5 mm wide. The largest prey animal was a heteropteran nymph (Fig. 3f) with a body 2.76 mm long and 1.24 mm wide. The other prey taxonomic groups were about 1–2 mm long. The taxonomic groups of Cladocera and Copepoda were the most diverse, with 9 and 7 determined different members of these families. The identified prey organisms are representatives of all movement groups, that is, substrate bound species (e.g. Pulmonata), grazers with swimming abilities (Ephemeroptera, Ostracoda, some Cladocera) as well as slow swimmers (e.g. some Cladocera, planktonic insect larvae, e.g. the ambush predator *Chaoborus* (Burrows and Dorosenko 2014), Hydrachnidia) and fast swimmers (Copepoda) (Fig. 5). The most common prey taxonomic group were Crustacea, they represent 44–77% of the prey items at all microsites.

We also found traps that contained more than just one prey organism, that is, 4 traps at GER and 12 traps in CZ. At the GER site, three traps contained free swimming prey (Hydrachnidia, *Daphnia*, Copepoda), and one trap contained a grazer (*Simocephalus*) together with free-swimming prey (a copepod). At the CZ site we found two snap-traps with three prey items. Prey content was either composed exclusively of grazers or free-swimming prey or both groups. Both traps with three items contained a mixture of grazers and free-swimmers.

Comparing the prey spectra of all microsites (Fig. 4), similarities on a high taxonomic level become obvious. On the other hand, all prey spectra are individual and not identical on the family or genus level. The most abundant taxonomic groups are Hydrachnidia (CZ1), Copepoda (CZ4, GER), Cladocera (CZ3, 5_June_, 7), Ostracoda (CZ5_August_, 2, 6), or Pulmonata (GER). Except for sites CZ4 and GER, more than half of the prey items are therefore Crustacea (up to 77%, CZ2). The representatives of Cladocera were differently distributed: Copepoda occurred at all sites, Ostracoda were only missing at GER. Cladocera were not found in the sampling at CZ4. At all sites, insect larvae, Pulmonata, or other prey items represent the rest of the prey. Just at GER, Pulmonata represent almost a third of the prey content (29%).

Considering the movement types of the above-mentioned taxa, at most microsites, grazer is the prevalent prey movement type. Mostly about 50% of the prey belong to this category. At CZ5_August_ as well as CZ7, even 69% and 64% are grazers, respectively. At GER, only 9% are grazers. Also, at CZ4, a comparatively small proportion are grazers (28%). At these microsites, substrate-bound species are quite well represented (23% at CZ4, 34% at GER). As Cladocera or Ostracoda occurred at all microsites, the group of slow free-swimming prey is represented at all microsites at 15% (CZ5_August_) to 40% (CZ2). Furthermore, fast free-swimming prey is also represented at all microsites with on average lower abundances of 8% (CZ1-7) to 31% (GER).

#### Temporal prey variation

Prey spectra identified from the same microsite but at different times of the year 2017 (CZ5_June_ and CZ5_August_) differed only in the partition patterns of taxonomic groups (Fig. 4). Except for Ephemeroptera larvae, which were found only in August and with a proportion of 21%, all prey taxonomic groups occurred during both sampling events. We observe a difference in the proportion of Cladocera that contained 45% in the June sample and 24% in the August sample. This difference attributed to the emergence of 21% of Ephemeroptera larvae and an increase of ostracods (15–35%). The proportion of Copepoda (15%) did not differ between June and August 2017. A shift in the taxonomic partition of prey organisms is also observed for Nematocera and Ostracoda which, in total, account for more than one-third of all taxonomic groups of both sampling events. While Nematocera are a quite common prey in June (20%), their proportion is reduced to 4% in August. In contrast, ostracods, which make up 15% of the prey in June, are prevalent in August (35%). Other prey taxonomic groups only account to 5% in June and 1% in August.

Following the strong reduction of Cladocera (often slow free-swimming prey) and the strong increase of Ephemeroptera (grazers), an increase of the proportion of grazers from 50% in June to 69% in August is observed at CZ5. Accordingly, the amount of fast free-swimming prey decreases from 20% to 15%, and also the amount of slow free-swimming prey. Substrate-bound prey was not found in June or August.

#### Differences in prey spectra between Czech and German sites

The combined prey spectrum from the Czech microsites from pooled sampling events CZ1–CZ7 (CZ_Total_, n_traps_=411) has an almost four times higher taxonomic diversity than the single spectrum from the German site (GER, n_traps_=34) (Fig. 4). In the CZ_Total_ traps we found 38 different prey taxa, in the samples from GER we recorded 9 different prey taxa (Table 1). Also compared to single Czech microsites, the GER microsite is still quite rare on taxa. Only at CZ5_June_, we found less taxa (7 taxa). At the other microsites, we found up to 20 different taxa in the traps. At all sites, the broadest prey range was found in the taxonomic groups Crustacea and Insecta. Hence, the overall prey spectra of the GER and CZ_Total_ sites are qualitatively comparable on a low taxonomic level (Fig. 4), but the quantities in prey differ noticeably. At both GER and CZ_Total_, roughly 40% of the prey is composed of cladocerans and copepods. While in the Wahner Heide, 18% of all filled traps contained Cladocera, 16% of the CZ_Total_ traps were filled with Copepoda. A considerable amount of Hydrachnidia was found in traps of both sites (CZ_Total_ traps: 9%, GER traps: 16%). While a quarter of the CZ_Total_ traps contained Ostracoda, this taxonomic group was not found at all in GER traps. Instead, GER traps predominantly contained snails (29%), especially of the family Physidae. Mollusca were present in just 2% of the CZ_Total_ traps. Ten percent of the CZ_Total_ traps contained other prey or matter (mainly insect larval stages, one amphibian egg, and one plant part). For the GER traps, apart from three insect larval stages, no other than the above-mentioned prey items were recorded.

**Table 1:** Trap contents of pooled plants from CZ and GER

Taxon list for pooled sampling events							
Group	Order	Familiy	Genus	Species epithet	Comment(s)	Movement type	Micro site(s)
Acari	Hydrachnidia	−	*−*	*−*	−	Slow free-swimming	GER
Acari	Hydrachnidia	Arrenuridae	*Arrenurus*	*cuspidator*	−	Slow free-swimming	GER
Acari	Hydrachnidia	Arrenuridae	*Arrenurus*	*globator*	−	Slow free-swimming	CZ1/2/3/5_June_/6
Acari	Hydrachnidia	Hydrachnidae	*Hydrachna*	*globosa*	−	Slow free-swimming	CZ6
Acari	Hydrachnidia	Hydrodromidae	*Hydrodroma*	*despiciens*	−	Slow free-swimming	CZ1/6
Acari	Hydrachnidia	Limnesiidae	*Limnesia*	*fulgida*	−	Slow free-swimming	CZ3
Acari	Hydrachnidia	Pionidae	*Piona*	*−*	−	Slow free-swimming	CZ1
Amphibia	Urodela	Salamandridae	*Lissotriton*	*−*	Egg	Not applicable	CZ1
Crustacea	Cladocera	Daphniidae	*−*	*−*	−	Slow free-swimming	CZ1/3/5_June_/5_August_/7
Crustacea	Cladocera	Daphniidae	*Acroperus*	*harpae*	−	Grazer	CZ1
Crustacea	Cladocera	Daphniidae	*Alona*	*−*	−	Grazer	CZ3
Crustacea	Cladocera	Daphniidae	*Alona*	*rectangula*	−	Grazer	CZ4
Crustacea	Cladocera	Daphniidae	*Ceriodaphnia*	*reticulata*	−	Slow free-swimming	CZ2/5_August_
Crustacea	Cladocera	Daphniidae	*Daphnia*	*−*	−	Slow free-swimming	GER
Crustacea	Cladocera	Daphniidae	*Daphnia*	*pulex*	−	Slow free-swimming	CZ6
Crustacea	Cladocera	Daphniidae	*Simocephalus*	*expinosus*	−	Grazer	CZ1/3/5_June_/5_August_/6/7/GER
Crustacea	Cladocera	Daphniidae	*Simocephalus*	*vetulus*	−	Grazer	CZ7
Crustacea	Copepoda	−	*−*	*−*	−		CZ1-7/GER
Crustacea	Copepoda	−	*−*	*−*	Eggs	Not applicable	CZ3
Crustacea	Copepoda	Cyclopidae	*Diacyclops*	*bicuspidatus*	−	Fast free-swimming	CZ4
Crustacea	Copepoda	Cyclopidae	*Eucyclops*	*−*	−	Fast free-swimming	CZ3/4/6
Crustacea	Copepoda	Cyclopidae	*Eucyclops*	*macrurus*	−	Fast free-swimming	CZ2/3/7
Crustacea	Copepoda	Cyclopidae	*Macrocyclops*	*albidus*	−	Fast free-swimming	CZ2/3
Crustacea	Copepoda	Cyclopidae	*Macrocyclops*	*fuscus*	−	Fast free-swimming	CZ6
Crustacea	Copepoda	Cyclopidae	*Megacyclops*	*viridis*	−	Fast free-swimming	CZ3/5_August_/7
Crustacea	Copepoda	Cyclopidae	*Thermocyclops*	*oithonoides*	−	Fast free-swimming	GER
Crustacea	Ostracoda	−	*−*	*−*	−	Grazer	CZ1-7
Crustacea	Ostracoda	Candonidae	*Candona*	*candida*	−	Grazer	CZ2/3/4/5_June_
Crustacea	Ostracoda	Candonidae	*Cyclocypris*	*laevis*	−	Grazer	CZ2/5_August_/6/7
Crustacea	Ostracoda	Cyprididae	*Herpetocypris*	*reptans*	−	Grazer	CZ2/4/5_August_/6/7
Insecta	Coleoptera	Dytiscidae	*Dytiscus*	*marginalis*	Larvae	Slow free-swimming	CZ3/4
Insecta	Ephemeroptera	−	*−*	*−*	Larvae	Grazer	CZ1/3/4/5_August_/7
Insecta	Heteroptera	Notonectidae	*Notonecta*	*−*	Nymph	Slow free-swimming	CZ2/4/6/GER
Insecta	Hymenoptera	−	−	−	Adult, accidentally?	Not applicable	GER
Insecta	Nematocera	−	*−*	*−*	Larvae	Slow free-swimming	CZ2/3/4/5_June_/5_August_
Insecta	Nematocera	Chaoboridae	*Chaoborus*	*−*	Larvae	Slow free-swimming	CZ5_August_/7
Insecta	Nematocera	Chironomidae	−	−	−	Slow free-swimming	CZ5_August_
Insecta	Nematocera	Sialidae	−	−	Larvae	Slow free-swimming	CZ2
Insecta	Odonata	−	−	−	Larvae	Grazer	CZ3/4
Insecta	Trichoptera	−	−	−	Larvae	Grazer	CZ4
Mollusca	Pulmonata	Physidae	−	−	−	Substrate-bound	CZ1/2/7/GER
Mollusca	Pulmonata	Planorbidae	−	−	−	Substrate-bound	CZ3/4
Plant	parts	−	−	−	−	Not applicable	CZ7

*Notes*: Dashes indicate that a more exact identification on family, genus, or species level was not possible due to the process of digestion or—in the case of larvae—the developmental stage. Movement types are given as used for the graphics.

To exclude a bias introduced by the various sampling periods, we also compared the German site (sampled May 29, 2017) with just these Czech samples originating within a more or less similar sampling period (CZ1-5_June_, June 10–11, 2017) (Fig. 4). By constraining the Czech samples to these sampling dates, the total number of investigated filled traps for this analysis was 256. Nevertheless, the proportions of prey changed just marginally. Copepoda were found in 18% (instead of 16%), Ostracoda in 24% (instead of 26%) of the traps. The partition of Hydrachnidia (11%) and Pulmonata (4%) changed by 2% each, Insecta occurred more often in earlier months (18%, instead of 10%). The median trap sizes for this sampling period are 3.49 mm for CZ traps and 2.81 mm for GER traps.

At the German and Czech sites, the abundance of slow free-swimming prey is almost constant (Fig. 5; 30% GER, 29% CZ_Total_). While at GER, we found more substrate-bound prey (34%), Czech traps contained mostly grazers (48%). Fast free-swimming prey is represented with 16% (GER) and 31% (CZ_Total_).

#### Correlations between trap size and prey size, taxa, and movement behavior

As we found mainly already partly digested prey in the traps, a reliable determination of prey size and, hence, an immediate testing of a correlation of accurate prey size and trap size was not possible. Therefore, we defined prey categories in ascending order of average size (“Plant parts”, “Copepoda eggs”, “Hydrachnidia”, “Ostracoda”, “Copepoda”, “Lissotriton egg”, “Daphniidae”, “Insect wing”, “Nematocera”, “Insect larvae”, “Insect”, “Heteroptera”, “Notonectidae”, “Physidae”, “Planorbidae”) and tested them for a correlation with trap size categories (“XS”, “S”, “M”, “L”, “XL”). We found no correlation between the prey category and the trap size, as the calculated linear model gave an *R*-value of −0.00054 (*p *=* *0.33817). Additionally, we tested for differences in variation of prey spectra between the trap size categories but found no significant differences (Kruskal–Wallis test, *p *=* *0.9682).

**Fig. 6:**
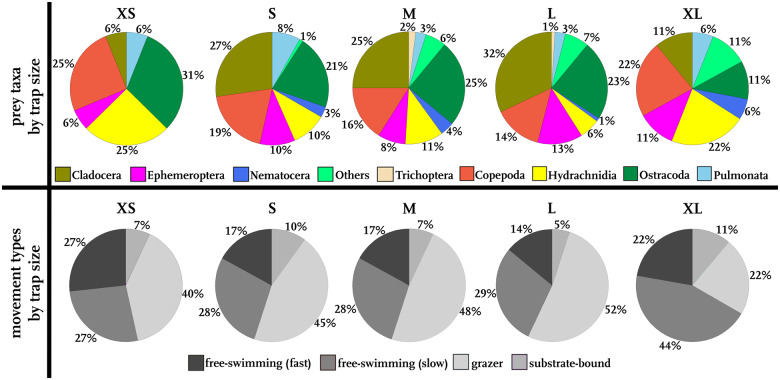
Prey taxa and movement types of prey, sorted by trap size. Given are the proportions of taxa in all traps of the sizes XS to XL and the movement types of the prey according to trap size. Data from all sites were pooled together.

Between the trap size categories, no considerable differences could be found in terms of the taxa composition (Fig. 6). Cladocera, Copepoda, Hydrachnidia, and Ostracoda are the main prey taxa. Especially Cladocera, Copepoda, and Ostracoda represent roughly two-thirds of all prey organisms in trap categories S (67%), M (66%), and L (69%). Hydrachnidia constitute around a fourth of the trap contents in trap size categories XS (25%) and XL (22%). Other prey taxa account for 1–7%, maximally 10–11% in all trap size categories.

In all trap sizes, grazers were found in large abundances (40–52%). Only in traps of the XL-category, grazers were not the main prey (22%). Here, slow free-swimming prey was most abundant (44%). Prey of this movement type constitutes 27–29% of the trap content. Substrate-bound prey like snails were found in 5–11% of the traps from all trap size categories. Fast free-swimming prey (Copepoda) were also present at similar abundancies of 14–27% throughout all trap size categories.

#### Correlations between water depth and trap content

With Spearman correlations, we found evidence for a strongly negative correlation between water depth of the microsite and Cladocera-abundance (r_Sp_=0.85, *p *<* *0.01). The deeper the water, the less Cladocerans we found. In all other taxa no evidence was found for any form of depth-dependency, neither a positive nor a negative significant correlation.

## Discussion

### Significance of the results

In the present study we investigated snap-trap sizes and the snap-trap contents of *A. vesiculosa* from one German and from seven Czech microsites. Although the sites are naturalized, the obtained results likely reflect the status of natural habitats (cf. Adamec 1995; Cross 2012) also because of the Central European origin of the introduced plants (see section “Methods”, study sites description). We here performed a comparative analysis of different field sites located in Central Europe, providing first detailed insights into the general prey spectrum of this carnivorous plant.

### Trap sizes

The measured snap-trap sizes match the reported length range of *A. vesiculosa* (cf. Cross 2012; Poppinga et al. 2018; Westermeier et al. 2018; Adamec 2018b). The differences measured between the sites and between sampling time points may be explained by differences in habitat quality (water chemistry, illumination), local weather conditions, and origin of the plants.

Moreover, the smallest snap-trap sizes found at the Wahner Heide site reflect unfavorable growth conditions for *A. vesiculosa*. Such a conclusion can further be supported by the very small population size, its permanent decline (five plants found later in 2017, one plant found in June 2018; personal observation), and short plants. Only under optimal conditions the largest traps can be formed (Adamec 1999, 2018b). Large traps allow catching small as well as big prey items, which appear to be additionally beneficial to *A. vesiculosa*, while small traps limit the prey spectra to smaller prey.

### Diversity of prey taxa

Among the prey taxa found, the taxonomic groups of Cladocera and Copepoda were the most diverse. Here, and also in the other taxonomic groups, more species are possibly captured, but could not be determined to family, genus or species level (Figs. 3 and 4; Table 1).

Overall, our analysis found a comparatively stable prey range that is comparable to the prey spectrum as reported by Akeret (1993). At the Swiss site, many Cladocera (38%), Ostracoda (16%), Copepoda (6%) as well as Ephemeroptera larvae (6%) were recorded and another considerable part of the prey contained dipteran larvae (28%) (cf. Fig. 4). Similar to the CZ microsites where we found 2% of Pulmonata, *A. vesiculosa* at Swiss microsites contained 6% pulmonates. Only the Wahner Heide microsite seems to be an exemption of this, as we here found 29% pulmonates. We anticipate that here Pulmonata are abundant so that they are captured by the traps when grazing for algae on *A. vesiculosa*.

In contrast, free-swimming prey covers more than 50% of *Aldrovanda*’s prey (Figs. 5 and 6). This is irrespective of the swimming velocities, as both fast swimmers and slow swimmers are found in the traps.

The most prominent prey organisms belong to the crustaceans represented by the taxonomic groups Cladocera, Ostracoda, and Copepoda. Furthermore, Mollusca from the families Planorbidae and Physidae, fall prey to *A. vesiculosa* traps. The small-sized mites of the taxonomic group Hydrachnidiae are also a common prey (9% in CZ_Total_ traps, 16% in GER). Insecta were found as prey items in similar proportions as water mites (8% in GER traps, 13% in CZ_Total_). Most Insecta found in the traps were larvae of either Ephemeroptera or chironomids. While chironomids were found in June and August, Ephemeroptera larvae where found only in August, with the exception of CZ1, where we also recorded five Ephemeroptera larvae in June. At Ptačí Blato 1st lagoon (CZ5_June_ and CZ5_August_), Ephemeroptera were only identified in the second sampling period. Possibly, adult Ephemeroptera emerged in June, mated and laid their eggs during June and July, leading to freshly hatched smaller larvae which then serve as prey for *A. vesiculosa* in August.

Overall, Insecta were the longest and largest prey items we recorded in the snap-traps. With 4.32 mm body length, a chironomid pupa was the longest prey item which only fitted into the traps by a bending of the abdomen to the ventral site. Indeed, Nematocera larvae were always found completely inside the respective traps, never with a body part (e.g. head) sticking out which was reported elsewhere and not only for *A. vesiculosa* (Cross 2012) but also for *Utricularia* (Brumpt 1925). Possibly, during escape trials, they winded and thereby pulled the rest of their body also into the trap. The second largest item found was a notonectid nymph, which was 2.76 mm long filling the trap substantially.

### Comparison of prey spectra between pooled Czech sites and the German site

Combined comparison of the of snap-trap contents of all Czech sites (CZ_Total_) with the German site (GER) revealed that the prey spectra are quite similar, that is, *A. vesiculosa* captures organisms from similar taxonomic groups, while only the individual proportions are variable. Especially, free-swimming prey (cladocerans and copepods) contributes to one-third of all found items. Both slow and fast free-swimming organisms make up 52–55% of all samples. The remaining prey consists of mainly substrate-bound organisms, like insects (larvae), ostracods, or snails.

We anticipate that the differences in prey spectra are explained by the relative abundance of taxa in the natural environment. These depend on biotic and abiotic factors of the respective field sites. For example, we found snails in 29% of the traps from Wahner Heide, but only in 2% of the traps from the Třeboňsko area. Unfortunately, no taxa lists are available for the investigated microsites; therefore, we could not proof the hypothesis that the found variance is due to the variable prey diversity, which nevertheless appears very likely. Furthermore, despite the sampling events May/June were timely very close together, a varying phenology may also influence the results, as Czech plants are located in a much more continental climate than traps from Wahner Heide (Germany). Therefore, the onset of summer may be earlier. Similarly, the comparatively high ostracod abundance at the pooled Czech sites may be due to that some water bodies (CZ2, 3, 5, 7) were very shallow in the dry 2017 season (Fig. 1). While the German *A. vesiculosa* population was found in an about 70 cm deep, artificial basin, the Czech sites’ depths are in many cases only 5–15 cm, sometimes up to 40 cm maximum. As ostracods are primary benthic organisms, the probability that they get in contact with *A. vesiculosa* plants floating on the surface is much higher in shallow waters. Nevertheless, no correlation between depth of water and ostracod proportion or other main prey categories’ (Copepoda and Hydrachnidia) proportions in the traps was found, except for Cladocera, for which we found an increasing abundance with decreasing depth (Supplementary Table 1). We expect this negative correlation to exist due to the niche choice of the found prey taxa. For example, Tessier and Welser(1991) described depth-specific distribution patterns of the Cladocera species occurring in lakes. In our study on shallower pools, most of the Cladocera prey taxa are either grazers (*Chydorus*, *Simocephalus*) or free-swimming organisms (*Ceriodaphnia*, *Daphnia*). As *A. vesiculosa* occurs only in the uppermost water layer, the probability that the above-mentioned prey taxa get in contact with this water layer and therefore with the traps, is much higher in shallow ponds. In general, such basins, in relation to the surface, provide less space to graze or swim in (see Hayashi and van der Kamp 2000). Apart from encounter probability reasons for this correlation, which could be valid also for other grazers and swimmers, cladocerans may also be more frequent in the respective shallow pools. At least during the sampling time, Cladocera may occur more commonly, probably due to biogeochemical conditions in these habitats. Such alterations with pool parameters have been described, for example, by Quiroga et al. (2013) or Simões et al. (2011).

### Temporal variation of prey spectra

Comparing temporal variability of (June and August) revealed that prey occurrence and abundance varies considerably and can change within short time spans. Apart from copepods, which accounted for 15% of the prey in both months, the rest of the prey spectrum was completely altered. While almost half (45%) of the prey comprised Cladocera in June, their amount decreased to 24%. Instead, ostracods became 20% more prevalent (35%). Furthermore, as already described in section “Diversity of prey taxa”, Ephemeroptera suddenly occurred in the prey spectrum, which is possibly due to their life cycle (Wise 1980). Some Ephemeroptera species lay their eggs in spring or early summer, resulting in small larvae in late summer, which later on do not fit in the traps anymore. The following spring, adults hatch, mate, and lay eggs again. *Aldrovandavesiculosa* inhabits mostly shallow waters which are prone to influences from the environment (heat, drought, rain). It is easily imaginable that this affects the prey community a lot, which is then mirrored in the prey spectra. Therefore, apart from geographical reasons for varying prey spectra, seasonal changes may explain a considerable amount of the variability found.

### Comparison to prey diversity from other geographical locations

*Aldrovanda* *vesiculosa* prey spectra from other regions than Europe (e.g. Australia) are completely missing until now. Some of the prey taxa found in our investigations were also reported from Japanese ponds inhabited by *A. vesiculosa* (Taira 2015). However, no investigation of the trap content and therefore the prey spectrum is reported from there. Except from Hydrachnidia, Ostracoda, and Pulmonata, which were not found in the ponds at the Japanese sites, two of the main prey taxonomic groups we found in our study (Copepoda, Cladocera) were also found there. It can be hypothesized that the water samples used for species determination in this investigation were acquired from the free water column, as just a few typical substrate-bound animals are listed, and also no insect larvae. In our analysis, we did not record Protozoa, Rotifera, and algae in our traps, and we expect these organisms to be too small for triggering the trapping mechanism mechanically. Hence, it can just be hypothesized that these animals would probably be by-catch. Nevertheless, as most of the species listed by Taira (2015) and as part of our snapshot prey analysis are widely distributed, they are very likely to appear in future prey spectra analyses also from other sites.

### Does *Aldrovanda* capture prey selectively or opportunistically?

Based on our results, we see no clear evidence for a specialization of *A. vesiculosa* to a certain prey type with respect to taxonomy, size, or behavior. Although a very broad range of prey sizes was recorded, very large prey including fish and tadpoles (cf. Darnowski et al. 2018) were not observed in this study, as they were missing at these sites during the sampling periods. While copepods are fast swimmers with measured velocities of 350 mm s^−1^ (Strickler 1975), Cladocera usually swim at intermediate velocities of about 5–25 mm s^−1^ (e.g. Wickramarathna et al. 2014; Heuschele et al. 2017). The other taxa can be regarded as either similar to Cladocera in their swimming velocities (e.g. Riessen 1982; Cooper et al. 1985; Burrows and Dorosenko 2014), are mostly bound to the substrate but also have swimming ability like ostracods, or are substrate-bound, the latter represented, for example, by Pulmonata. As all these movement groups fall prey to *A. vesiculosa*, we concluded that the plant has no preference for slow- or fast-moving prey and also no obvious preference for free-swimming or substrate-bound prey (Fig. 5). Nevertheless, at most sites, a majority of all prey items were grazers, which just simply might be due to their abundance in the investigated waters (which was not further investigated in this study). We expect that in particular the ostracods and snails graze on the plants (e.g. for algae) which eventually leads them into the traps and entails their capture. However, the existence of mechanical guiding structures as in *Utricularia* suction traps (Meyers and Strickler 1979) as well as a possible attraction role of the *A. vesiculosa* trigger hairs (Schell 2003) remain speculative. Especially for the not free-swimming prey organisms, it can be hypothesized that *A. vesiculosa* simply serves as substrate to graze on. Furthermore, snails could be attracted by the sinking and decaying older plant parts, as it may be an attractive food source.

As described earlier, Schell (2003) speculates that the trigger hairs of *A. vesiculosa* imitate filamentous algae as periphyton and, therefore, attract animals that feed on them. As most animals we found are relatively small, especially the snails, it appears rather unlikely that animals of this size are physically able to feed on larger filamentous algae that *A. vesiculosa* could imitate. Possibly, adult stages of snails feed on them, but these are too large to be captured.

Like the ostracods caught during grazing on the algae growing on the plants, it can be hypothesized that many capture events may occur coincidentally, as no preference for a special prey category was revealed. Furthermore, a specialized “broadband” prey attraction strategy, which targets at so many different organisms as observed in our investigated traps, appears rather unlikely. Instead, we suggest that animals move freely in the water or on the plants, when they just by chance get in between the trap’s halves and elicit the closing mechanism. Therefore, we can support the hypothesis by Akeret (1993) that many prey organisms stray into the traps accidentally. Furthermore, Akeret hypothesized also the non-swimming organisms like Pulmonata and insect larvae to serve as food source, which we can confirm based on the different digestion progresses we found for these organisms in our investigated traps. Similarly, notonectids and other insect larvae may be caught by moving on the plants or alternatively by unguided swimming movements, for example, during escape. Organisms that are predators themselves, like the captured juvenile *Notonecta*, may use the floating plants as stand for their hunt when they eventually get caught themselves.

We could not find any striking difference in prey species composition among small and large traps either (Fig. 6). Only in very large traps longer than 4 mm, the abundance of slow free-swimming prey increases. This may be due to a possibly reduced sensitivity to small prey like grazers with increasing size. Furthermore, traps of this size may be able to capture larger free-swimming prey, which in turn reduces the abundance of grazers relatively. Apparently, all animals that fit in the traps are caught, and sometimes even larger prey is clamped between the lobes (and probably partially digested) (cf. Cross 2012; Darnowski et al. 2018). Of course, that excludes very big prey from very small traps, but does not specify big traps to catch only comparatively big prey items. However, after prey capture, the traps of *Aldrovanda* perform a narrowing movement until a sickle-shaped cavity (*sensu*Ashida 1934) is formed (Fig. 3b), where digestion takes place. It can be concluded, for example, from Figs. 3d and 3f that prey may also be trapped between the marginal trap lobe parts. It remains to be evaluated if such clamped prey can be (fully or partly) digested by the plant, or if there occurs critical leakage of the enzymatic cocktail produced for digestion.

Depending on the prey diversity the waters maintain, *A. vesiculosa* apparently can exploit various food sources, making it a generalist predator. Nevertheless, from the quantitative point of view, the plants may not be a very efficient predator. As follows from the counts of sampled *A. vesiculosa* plants at all microsites, on average only 1–6 filled traps occurred per plant, out of the total ca. 45 traps in the sampling zone per plant at the German site and ca. 83 traps at the Czech sites. Thus, at maximum on average only ca. 1–6.6% of all functional traps captured prey. Similar low values of 5–8% reported also Adamec and Kovářová (2006) for CZ4 and CZ5 with that the higher rate was attained at more a fertile site. Whereas the German population contained only 15 plants exhibiting a low density, the *Aldrovanda* density at the Czech sites was much higher, which also points toward a greater grazing pressure and, thus, lower capture efficiency. However, the majority of traps was filled with prey in a growth experiment with abundant feeding on ostracods (Adamec et al. 2010). Therefore, prey availability (abundance) rather than prey quality presumably decides on the success of prey capture. In this regard it is important to keep in mind that, in our study, we were not able to compare the prey spectra of *Aldrovanda* (as analyzed) to the species spectra (potential prey) available at the respective microsites. Therefore, our interpretation and discussion of trapping efficiency must be taken with some caution.

After all, we expect *A. vesiculosa* to be principally a quite successful predator for many prey organisms (Cross et al. 2015), and by this our results are contradicting Akeret (1993). Therefore, we also expect the actual prey spectra to be explained by site-specific prey abundances, similar to the terrestrial carnivorous sundew *Drosera rotundifolia* (Cook et al. 2018). Indeed, the capability to capture a high diversity of different prey taxa may facilitate *A. vesiculosa* to thrive in small, fragmented, and also highly diverse habitats where a more or less strict prey specialization might otherwise be a selective disadvantage.

As we found no conspicuous amounts of detritus and algae inside the traps investigated, we hypothesize that such organic matter may play no (significant) role in *A. vesiculosa* nourishment, in contrast to aquatic bladderworts which can be considered as partially vegetarian (Mette et al. 2000; Alkhalaf et al. 2009; Koller-Peroutka et al. 2015; Ellwood et al. 2018).

Also, the closest *A. vesiculosa* relative, the terrestrial Venus flytrap (*Dionaea muscipula*), was shown to capture opportunistically rather that selectively in field investigations (Hutchens and Luken 2009, 2015), although this is in contrast to ecological models by other authors (Gibson and Waller 2009; Lehtinen 2018). Interestingly, the question as to why and how snap-traps evolved in two species with different life forms (terrestrial, aquatic) with different snap-trap mechanics (Forterre et al. 2005; Poppinga and Joyeux 2011; Westermeier et al. 2018) remains unsolved, as both mechanisms may partly operate in both media (at least for *Dionaea*, see Poppinga et al. 2016a) and do not exhibit specialized capture of certain prey taxa.

### Future perspectives

*Aldrovanda vesiculosa* seems to have a very variable prey spectrum and is able to catch in its snap-traps whatever prey be present at the sites. It is therefore shown to be very opportunistic. To further support this hypothesis, the prey diversity estimated at the sites requires comparison with taxa proportions available at the site whether they are reflected proportionally in the trap content or a certain prey group is overrepresented.

Considering conservational issues, it is interesting how flexible *A. vesiculosa* seems to be regarding the available prey diversity. As the taxa of the determined prey spectra are almost ubiquitous, from that point of view, possible *A. vesiculosa* sites should not be rare. Nevertheless, *A. vesiculosa* is outcompeted by other plants possibly due to its very narrow range for abiotic factors (see Adamec 2018b).

Additionally, similar comparative studies as presented here should be conducted in natural populations of *A. vesiculosa*, not only in Europe but also in other continents. These studies should include the comparison of potential prey availability in habitats with the captured prey. Such large-scale prey spectra comparisons of a single carnivorous plant species (with different ecotypes) should shed light on the efficiency and adaptability or functional similarity of the trapping system. It may be further worth testing for possible correlations of the trap age versus a trap content (cf. Hatcher and Hart 2014). Laboratory feeding experiments on selected prey taxa should determine the absolute capturing preferences of these taxa. Not only are older traps bigger, but also trap closing duration or sensitivity may vary (cf. Ashida 1934; Westermeier et al. 2018). For *Dionaea*, it is known that for triggering the shutting movement at least two stimuli within 20–30 s are required (Williams and Bennett 1982; Hodick and Sievers 1988). For *A. vesiculosa*, no detailed analyses have been performed yet in this context, and it remains conceivable that age-dependent and/or trap-specific differences exist, leading to different trap reaction/closure times and, thereby, prey spectra. However, for analysis, it remains problematic that the digestion state is hard to distinguish. This hinders to determine the time point of ingestion, that is, just-opened or aged.

## Supplementary Material

Supplementary Data
