# Supplementary material for: Comparative Prey Spectra Analyses on the Endangered Aquatic Carnivorous Waterwheel Plant (Aldrovanda vesiculosa, Droseraceae) at Several Naturalized Microsites in the Czech Republic and Germany
Source: Integr Org Biol. 2019 Mar 25;1(1):oby012. doi: 10.1093/iob/oby012 (PMC7671111; doi:10.1093/iob/oby012)
Supplement: Supplementary Data [file oby012_supp.pdf]

Supplementary Table 1: Correlation values for the analysis of trap vs. prey size and the analysis prey item vs. water depth of the microsite.

	test	R^2/r_{Sp}	p
Prey size vs. trap size	Linear model	-0.00054	0.33817
Prey variance vs. trap size	Kruskal Wallis	-	0.9682
Water depth vs. prey variance (Cladocera)	Spearman correlation	-0.8539	0.007
Water depth vs. prey variance (other taxa)	Spearman correlation	-0.034-0.531	0.1412-0.931
